# Preparation and Characterization of Al/HTPB Composite for High Energetic Materials

**DOI:** 10.3390/nano10112222

**Published:** 2020-11-08

**Authors:** Alexander Vorozhtsov, Marat Lerner, Nikolay Rodkevich, Sergei Sokolov, Elizaveta Perchatkina, Christian Paravan

**Affiliations:** 1 National Research Tomsk State University, Lenin Avenue, 36, Tomsk 634050, Russia; abv1953@mail.ru (A.V.); lerner@ispms.tsc.ru (M.L.); perchatkinae@mail.ru (E.P.); 2 Institute of Physics Strength and Material Science SB RAS, Avenue Akademicheskii, 2/4, Tomsk 634055, Russia; ngradk@ispms.tsc.ru; 3 Department of Aerospace Science and Technology, Politecnico di Milano, 34 via LaMasa, I-20156 Milan, Italy; christian.paravan@polimi.it

**Keywords:** HTPB, aluminum nanopowders, solid propellants, burning rate, coated aluminum

## Abstract

Nanosized Al (nAl) powders offer increased reactivity than the conventional micron-sized counterpart, thanks to their reduced size and increased specific surface area. While desirable from the combustion viewpoint, this high reactivity comes at the cost of difficult handling and implementation of the nanosized powders in preparations. The coating with hydroxyl-terminated polybutadiene (HTPB) is proposed to improve powder handling and ease of use of nAl and to limit its sensitivity to aging. The nAl/HTPB composite can be an intermediate product for the subsequent manufacturing of mixed high-energy materials while maintaining the qualities and advantages of nAl. In this work, experimental studies of the high-energy mixture nAl/HTPB are carried out. The investigated materials include two composites: nAl (90 wt.%) + HTPB (10 wt.%) and nAl (80 wt.%) + HTPB (20 wt.%). Thermogravimetric analysis (TGA) is performed from 30 to 1000 °C at slow heating rate (10 °C/min) in inert (Ar) and oxidizing (air) environment. The combustion characteristics of propellant formulations loaded with conventional and HTPB-coated nAl are analyzed and discussed. Results show the increased burning rate performance of nAl/HTPB-loaded propellants over the counterpart loaded with micron-sized Al.

## 1. Introduction

The common composition of mixed high-energy material (HEM) as heterogeneous solid propellants is based on three main ingredients: an oxidizing agent, an organic binder, and a high energy density fuel. Ammonium perchlorate (AP) or ammonium nitrate (AN) are typically used as oxidizer, low-molecular-weight polybutadiene (usually cured by isocyanates) is a common binder, while metal powders are considered as fuels. Aluminum powder is the most readily available, intensively studied and efficient metallic fuel in various HEM formulations. As the aluminum particle size decreases, the ignition temperature and combustion time decrease [1,2]. In solid propellants, the replacement of micron-sized Al powders (µAl) with nanosized aluminum powders (nAl) leads to an increase in the burning rate and a decrease in the size of the condensed combustion products (aggregates/agglomerates) in the near-surface zone [3]. An analysis of the effects of Al powder size reduction on the burning rate of solid propellants and on the condensed combustion products formation is reported in [4]. In this latter study, the burning rate of solid propellants loaded with Al particles with size of 30 μm and 170 nm was measured [4]. Under the investigated conditions, the burning rate was almost doubled when µAl powders were replaced by nAl [4].

Nanosized metal powders are characterized by very high specific surface area. Nanoparticles have different chemical and physical properties when compared to micron sized powders. Due to their increased reactivity, nanopowders are very attractive ingredients for HEM formulations [5,6,7,8,9,10,11,12].

Despite the obvious advantages for inclusion nAl powders in the HEM formulations (i.e., faster reaction rates), there are some disadvantages that limit their use. Nanosized Al is more sensitive than the micron-sized counterpart to the influence of oxidizing or corrosive environment due to the large specific surface area [13,14]. This increased sensitivity to the environment may lead to significant active aluminum content losses during nAl storage [14]. As a consequence, nAl could release less energy during the combustion, thus decreasing the combustion performance of HEMs loaded with it. To suppress/limit the nAl aging during storage, the nanoparticles surface (typically passivated by Al_2_O_3_) can be coated by a protective layer [15,16,17]. The encapsulation of particles with hydrocarbon/fluorohydrocarbon coatings has shown the promising results in the limitation of aging process of nAl powder [18]. Hydroxyl-terminated polybutadiene (HTPB) is a suitable material for nAl capping when solid propellant applications are targeted, since it is a common binder in composite formulations for solid rocket propulsion [19,20]. The HTPB offers very low glass transition temperature, relatively low viscosity, high combustion heat and, once cured, high mechanical properties of the final product even at high powder filling ratios.

In this paper the use of HTPB as a protective coating for nAl is discussed. The nAl powder coating process includes the use of acetylacetone together with HTPB. In the proposed strategy the acetylacetone is applied on the particle first. Thus, protective organic hydrophobic layer is created on the surface of the nanoparticles. Then, HTPB is sorbed on the hydrophobic surface of the nanoparticles increasing the compatibility of the nAl with binder and propellant components, preventing a decrease in the content of active aluminum due to aging, and can also simplify propellant production and casting [18]. For powders added to the propellant, the same polymer is used as a binder as for coating the particles. However, when mixing the nanopowders and polymer, the viscosity of the mixture increases drastically, slightly decreasing with continued mixing. Since the mixture has a high viscosity, its preparation requires a lot of time and energy. To overcome this problem, an important technological stage is the preliminary preparation of pastes based on metal nanopowders and polymer binders, followed by their inclusion in the HEM formulation.

The propellant burning rate is a key parameter for the design of a solid rocket motor and, in particular for the thrust level [21]. Together with the oxidizer decomposition and sublimation, and the metal combustion mechanism, HTPB degradation is one of the significant stages of the propellant burning [22]. Thus, the study of the effect of the propellant components on the process of destruction of HTPB is an urgent task in the field of propellant improvement.

The main aim of this work is the experimental study the characteristics of different nAl-based HTPB-containing pastes (nAl/HTPB). Various pastes were prepared, the difference between them being the nAl-to-HTPB ratio. The work focuses on two nAl/HTPB (10 wt.% and 20 wt.%) that are contrasted in terms of their characteristics and effects on the burning behavior of solid propellants loaded with them.

## 2. Materials and Methods

Aluminum nanopowder (nAl) used in present work was produced by electric explosion of wire (EEW) method [23,24].

Hydroxyl-terminated polybutadiene (HTPB R45), acetylacetone of analytical grade and mineral spirit (used as solvents) with boiling point in the range of 70–100 °C were used without any further purification.

Ammonium perchlorate (AP) used for the preparation of the propellant formulations was used in the form of fine and coarse powders, the fractional composition of which is shown in Table 1.

Microstructure and morphology of the aluminum nanopowders were captured by a JEM-100 CXII transmission electron microscope (TEM, JEOL Ltd., Tokyo, Japan) for both pristine air-passivated and HTPB-coated powders. Active aluminum content (C_Al_) was determined by volumetric method. The content was estimated by the evolution of the hydrogen released by the powder reaction with a 5 M NaOH solution [25].

Aluminum nanopowders specific surface area was determined by nitrogen adsorption/desorption by the Brunauer-Emmett-Teller (BET) method with a SORBTOMETR-M (Katakon, Russia) surface area analyzer. A specimen mass of 50 mg was used for the measurements. Specific surface area samples had been heated at 120 °C for 30 min before the surface area measurement was taken. The average particle diameter based on the specific surface area was calculated assuming a particle size distribution of uniform spheres, as:

(1) $$d=\frac{6000}{\rho S}$$

where *d*—nanoparticle diameter, nm, *ρ*—particle density, g/cm^3^, *S*—BET specific surface area, m^2^/g [26].

The reactivity and stability of the powder at various temperatures and heating rates was studied with use of thermogravimetric analysis (TGA) and differential thermal analysis (DTA) Seiko Exstar 6000 (Seiko Instruments Inc., Chiba, Japan). The samples of 5–10 mg weight were heated from ambient temperature to 1273 K at heating rate 10 °C/min in air atmosphere. The TGA/differential scanning calorimetry (DSC) experiments in Ar were performed by a Netzsch F5 Jupiter STA analyzer (NETZSCH-Gerätebau GmbH, Selb, Germany) under the same conditions of the DTA scans.

To prepare the nAl/HTPB composition, for example with HTPB content of 10 wt.% (nAl-H10), 250 mL of solvent, 45.0 g of nAl powder, and 0.225 g of acetylacetone were used. The amount of acetylacetone was 0.5% of the weight of the bare nanopowder. The resulting mixture was stirred with a high shear homogenizer HG-15D (Daihan Scientific, Seoul, Korea) at 5000 rpm for 30 min. Then a HTPB-based solution (5.0 g HTPB in 50 mL of solvent) was added to the mixture and was then stirred for another 30 min. The solvent was removed from the mixture using an IKA 10 RV (IKA^®^-Werke GmbH & Co. KG, Staufen im Breisgau, Germany) rotary evaporator; the resulting product was dried at a pressure of 1.33 mbar for 16 h. The content of components in the samples are listed in Table 2.

Resonant acoustic mixing (LabRAM I apparatus, Resodyn Acoustic Mixers, Butte, MT, USA) was used to uniformly disperse the propellant ingredients [27].

The binder was prepared starting from HTPB R45 resin cured with isophorone diisocyanate (IPDI). The curing ratio of the binder ([-NCO]/[-OH]) was 1.04. Dioctyl adipate (DOA) was used as a plasticizer, while dibutyltin diacetate was added (in excess) to the formulation as curing catalyst. For a comparative assessment the propellant formulation was prepared with the initial nAl powder as presented in Table 3. The nAl mass fraction in the tested formulation is the same on a molar basis. For all the tested formulations, the coarse-to-fine AP ratio is 65:10.

**Figure 1:**
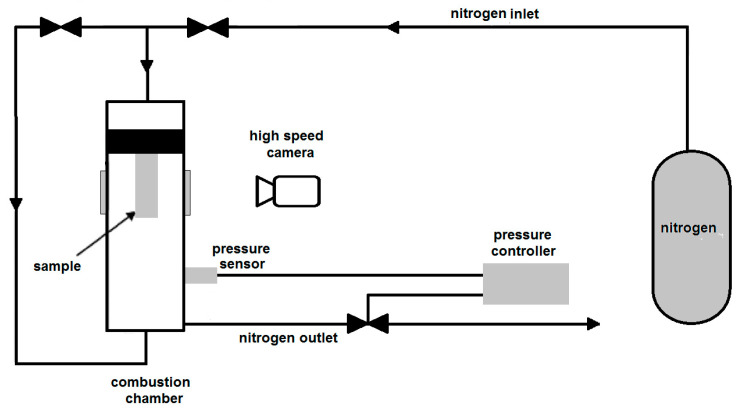
Schematic diagram of the lab-scale strand burner for *r_b_* determination setup.

The propellant burning rate (*r_b_*) was determined from tests carried out in a laboratory combustion chamber equipped with windows for combustion process video recording. The tested samples are cut into parallelepipeds (4 × 4 × 30 mm). The lateral surface of the samples is inhibited to provide a one-dimensional regression of the combustion surface. A simplified scheme of the experimental setup is given in Figure 1. Nitrogen is used for the combustion chamber pressurization, and to prevent combustion products smoke from inhibiting the visualization. Quasi-steady chamber pressure is granted by electrovalves controlled by a digital pressure regulator. The r_b_ value is determined using by a proprietary software. The latter enables the regressing surface tracking during the combustion, thus providing the quasi-steady burning rate. For a given propellant, experimental results are reduced according to the standard Vielle’s law [28]:

r_b_ = ap^n^ (2)

In the Equation (2), the *r_b_* is typically expressed in mm/s, with *p* in bar.

Characterization of the nanopowders developed by Tomsk State University (TSU, Tomsk, Russia) is carried out in cooperation with the Space Propulsion Laboratory of the Politecnico di Milano (SPLAB-POLIMI, Milan, Italy). Investigation of the combustion of the propellants containing nanopowders is carried out by SPLAB-POLIMI.

## 3. Results and Discussion

In an earlier stage of the work, a number of nAl/HTPB composites were prepared. Manufactured materials featured an HTPB content in the range 10 to 50 wt.%. The composites with HTPB content of 10 wt.% and 20 wt.% are disperse solids, while for HTPB mass fractions >20%, the obtained materials are highly viscous masses of difficult handling. Thus, activities concentrated on nAl100-H10 and nAl100-H20.

### 3.1. nAl Characterization

**Figure 2:**
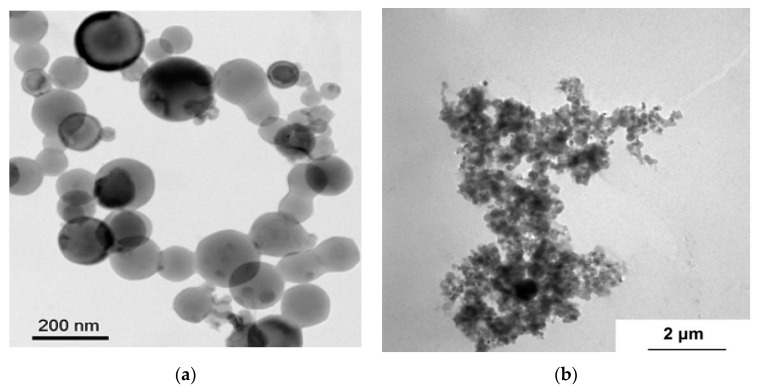
TEM images of the (**a**) pristine aluminum nanoparticles and (**b**) an aluminum nanoparticle cluster.

A TEM image of the pristine, air-passivated aluminum nanopowder is presented in Figure 2. The shape of the visible nanoparticles is predominantly spherical. The native amorphous oxide layer consisting of bayerite α-Al(OH)_3_ and γ-AlOOH boehmite [13] is on the surface of aluminum nanoparticles. Content of aluminum metal is usually about 90 wt.% [17,29]. Nanoparticles have a marked clustering tendency with formation of particles subject to cold cohesion reaching sizes up to hundreds of microns. The observed clusters are rather compact, yet the cold-cohesion particle-particle interactions are typically relatively weak, and clusters can be broken down (though reversibly) by mechanical stresses (e.g., ultrasound irradiation).

The content of active aluminum in the aluminum nanopowders used in the present work is (85.9 ± 0.8) wt.% as presented in Table 4. The BET—derived specific surface of the nanopowder is 12.6 ± 0.1 m^2^/g, corresponding to an average diameter of the nanoparticles [Equation (1)] of ~175 nm.

Table 4 shows C_Al_ data for all the tested powders. For the nAl/HTPB, the actual C_Al_ is compared to the expected value resulting from the pristine powder (nAl100) and the nominal coating mass fraction (10 wt.%, 20 wt.%). The difference between the actual and the expected values reported in the Table 4 is originated by the possible presence of (minor amounts of) residual solvent in the HTPB-containing pastes. From this point of view, this observation is supported by the slightly higher C_Al_ difference for nAl100-H20, which contains a higher HTPB mass fraction.

### 3.2. Characterization of the nAl/HTPB Pastes

**Figure 3:**
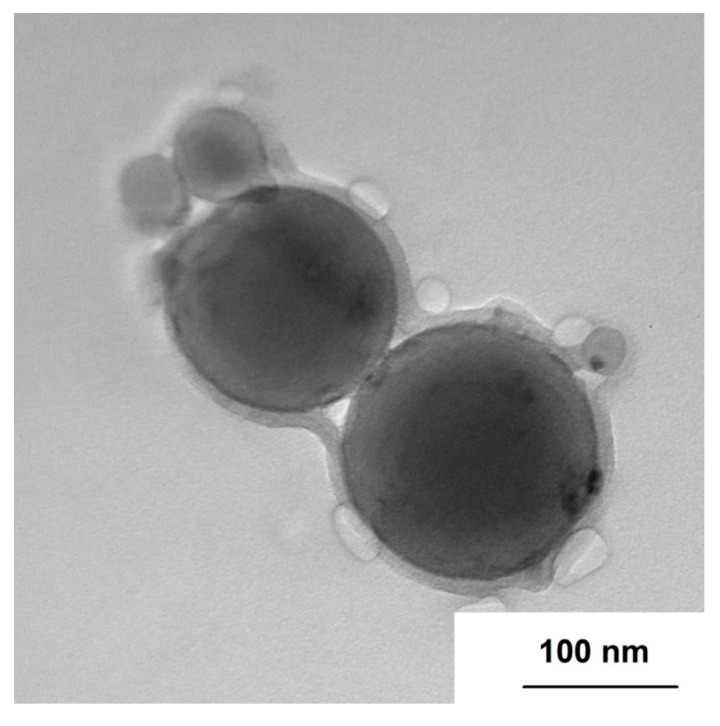
Aluminum nanoparticles coated with HTPB.

An image of the nAl100-H10 is reported in Figure 3, where HTPB is seen to form a continuous organic layer, partially sorbed on the surface of the nanoparticles or particle clusters. Data reported in the Table 4 suggest the absence of powder-HTPB reactions modifying the powder composition: when preparing the composite, neither the formation of soluble aluminum compounds occurs when interacting with an organic substance, nor the sorption of the solvent by the particles of the composite. The HTPB deposition at the particle surface provides a protective layer shielding the nanoparticle surface from interaction with the environment, thus offering an increased aging resistance [21]. At the same time, the HTPB deposition promotes particle clustering (see Figure 3, where separated particles are encapsulated by the HTPB layer) [21], yet the impact of this effect should be evaluated considering the powder mixing and dispersion in the HEM matrix.

### 3.3. Thermogravimetric Analysis of the Pastes

#### 3.3.1. Thermogravimetric Analysis of the Pastes in Ar

**Figure 4:**
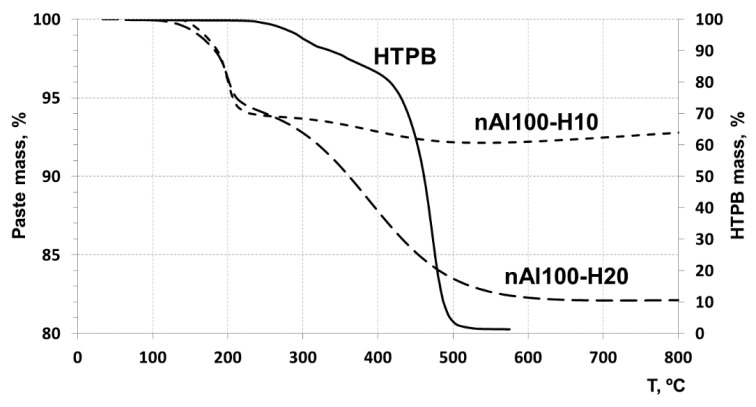
TGA of HTPB and nAl/HTPB pastes in Ar (10 °C/min).

The TGA plots of HTPB and nAl-HTPB samples are shown in Figure 4. Pristine, uncured HTPB can be seen to undergo two-stage degradation in an argon atmosphere. The first stage of the destruction starts at about 225 °C and continues up to about 325 °C with a weight loss of ~11% at this stage. The second stage of the thermal degradation proceeds in the temperature range from 325 to 525 °C with almost complete mass loss. The rate of the HTPB degradation in this second stage, based on the TGA curve slope, is higher than that at the first stage.

The thermal degradation of HTPB in the nAl/HTPB pastes proceeds as a two-stage process too. However, the first stage of the destruction begins at a lower temperature (~100 °C) and ends at about 210 °C. Weight loss at this stage is 7–8%. The weight loss at this stage is independent of the paste composition. The destruction of the polymer can be assumed to occur on the nanoparticle surface and its surface oxide layer is a destruction catalyst, since the destruction rate in this case is higher than that in the lack of contact with alumina, despite the destruction proceeds at a lower temperature.

The thermal degradation of HTPB in the second stage proceeds at a lower rate compared with that at the first stage, although at a higher temperature. The thermal degradation end occurs in the range 500–550 °C, which suggests that polymer pyrolysis at this stage proceeds outside of its contact with alumina of the aluminum nanoparticle and the polymer degradation mechanism does not change, with a lower destruction rate due to a lower onset decomposition temperature.

#### 3.3.2. Thermogravimetric Analysis of the Pastes in Air

**Figure 5:**
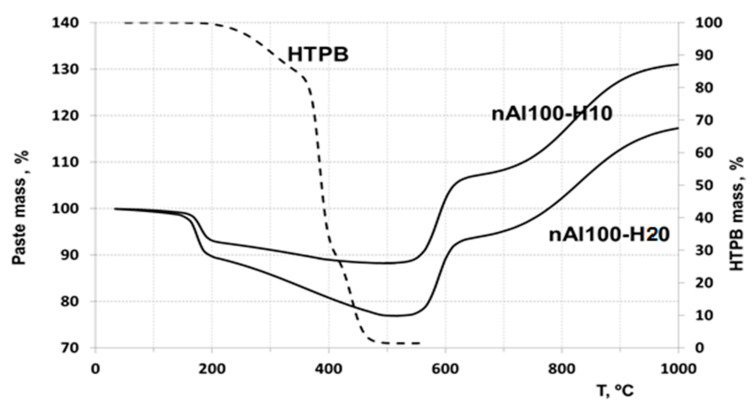
TGA of HTPB and nAl/HTPB pastes in air (10 °C/min).

TGA plots of HTPB and nAl/HTPB samples performed in air are shown in Figure 5.

Initial HTPB as can be seen from the plot undergoes two-stage mass-loss, as per the tests in Ar (Figure 4). The mass loss at the first stage of decomposition is insignificantly higher as compared to decomposition in Ar. The first stage of decomposition begins at about 200 °C and continues up to about 380 °C with a weight loss of ~7% at this stage. The second stage of reaction proceeds in the temperature range from 380 to 475 °C with almost complete weight loss. The rate of decomposition of HTPB at this stage, based on the TG curve slope is higher than that at the first stage.

The degradation/reaction of the HTPB applied on the surface of the nAl particles proceeds as in the Ar case in two-stages, however, the first stage of the decomposition begins at a lower temperature about 100 °C and ends at about 200 °C. Weight loss at this stage depends on the composition of the paste, weight loss being larger when increasing HTPB content. Above 200 °C the second stage of the destruction begins, with the completion to be about 500 °C. Aluminum nanoparticle oxidation begins at the temperature above 500 °C. In the temperature range 550–650 °C, oxidation of aluminum nanoparticles is observed, accompanied by their partial sintering [30]. Subsequent oxidation of aluminum is observed at temperature above 650 °C and continues at temperatures above 1000 °C.

### 3.4. Burning Rate

**Figure 6:**
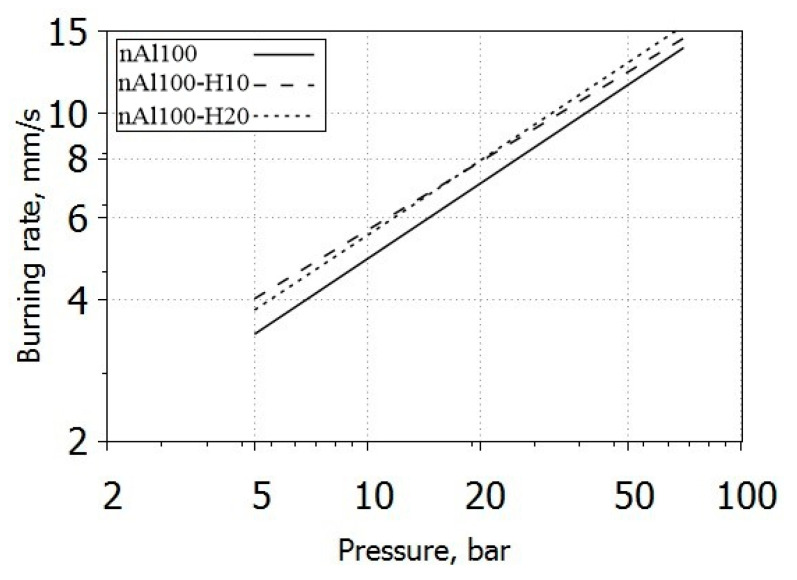
Burning rate of the propellants with aluminum nanopowders.

The propellant burning rate generally follows Vieille’s law as shown in Equation (2). Data fittings and burning rate values obtained from propellant tests are shown in Figure 6 and Table 5.

The data show the introduction of nAl/HTPB paste to provide an increase in the propellant burning rate. Compared to nAl100, the introduction of paste into the propellant formulation results in an increase in the rate coefficient *a* and a decrease in the value of the pressure exponent *n*. The burning rate increase suggest a better dispersion of the metal particles of the nAl/HTPB pastes in the propellant matrix. The value of pressure exponent for the propellants with introduced paste is somewhat lower compared with that for propellant with initial aluminum nanopowder. However, considering the interval of confidence on the ballistic exponent, its variations are relatively small (if any). This suggest the absence of changes in the combustion mechanism of the tested formulations.

## 4. Discussion

nAl/HTPB composites were prepared by wet mixing method. The preparation includes two stages. The first stage is the disruption of nAl particle clusters when stirring with acetylacetone and formation of the organic chemisorbed layer on the nanoparticle surface. This layer increases the hydrophobicity and compatibility with the polymer of the nanoparticle surface and facilitates the sorption of the HTPB molecules [31]. The second stage is the application of HTPB on the nanoparticle surface. TEM images show the homogeneous continuous layer of the polymer capping the nanoparticle/nanoparticles. The capping prevents direct contacts of the nanoparticles with each other (for separated particles), and hinders the penetration of oxidant molecules to the nanoparticle surface, which increases their stability during storage and processing. This effect provides the complete protection of the nanoparticles against environmental influences but retains their high reactivity which is demonstrated by nanoparticles slow heating rate oxidation and combustion tests [18], see Figure 5 and Table 5.

TGA analysis shows the lack of a chemical bond between the nanoparticle and HTPB molecules. Thermal degradation of HTPB during slow heating rate heating proceeds as intramolecular chemical process with minimal participation of oxygen. Only part of the HTPB molecules in contact with the nanoparticle surface is degraded at a lower temperature by the action of alumina layer as catalyst. So the HTPB destruction proceeds and aluminum oxidation sequential reactions occurring independently of each other. The thermal degradation of HTPB in the nAl/HTPB pastes at the second stage proceeds in the same temperature range as that of HTPB [31]. The degradation proceeding at this stage can be assumed to be noncatalytic process. The degradation process at this stage is suggested to proceed outside of the contact with alumina of the aluminum nanoparticle and the HTPB degradation mechanism is the same both for nAl/HTPB and HTPB.

The propellants with formulation comprising capped aluminum nanoparticles exhibit higher burning rate than the counterpart loaded with nAl100. Yet, the fundamentals of the burning process remain unchanged. Quantitative parameters of the burning law allow to consider the burning process to be faster and more stable when capped aluminum nanoparticles were added.

## 5. Conclusions

Different nAl/HTPB composites were prepared by a wet method. Of the prepared materials (HTPB mass fraction in the range 10 to 50 wt.%), two were selected for detailed analyses spanning from pre-burning characterizations to combustion analyses. The selection was made based on the availability of a disperse phase system with powder-like characteristics, and limited volability. In particular, the selected composites featured the following compositions (i) nAl100 (90 wt.%) + HTPB (10 wt.%) and (ii) nAl100 (80 wt.%) + HTPB (20 wt.%). In both materials acetylacetone served as compatibilizing agent for effective wetting of the nAl surface (Al_2_O_3_ from air passivation, with possibly, hydrated compounds) with HTPB. The nAl/HTPB composite can be an intermediate product for the subsequent manufacture of mixed high-energy materials while maintaining the quality and advantages of nAl. Capping of the nanoparticle surface with HTPB protects nanoparticles against environmental influence (i.e., material corruption due to ageing) and provides easier handling and manufacturing while granting good combustion performance as tesitifed by DTA-TGA scans and burning tests performed in a lab-scale burner. The slow heating rate, non-isothermal oxidation of the powders showed the effect of the nAl on the HTPB degradation process proceeding at lower temperature and with higher reaction rate than what is observed for the uncured polymer alone. On the other hand, no evidences of HTPB-aluminum interactions affecting the powder reactivity and oxidation mechanism were noticed. Thermal degradation effects were separately investigated in Ar and air to decouple the polymer pyrolysis effects from the composite oxidation mechanism.

The burning characteristics of the propellant formulations containing tested composites were analyzed. The inclusion of the composite in the propellant formulation provides faster burning rate with increasing burning stability at low pressure.

## Figures and Tables

**Table 1:** Ammonium perchlorate (AP) particle size distribution.

Parameter	*AP_coarse_*	*AP_fine_*
D_0.1_, µm	139 ± 1.39	1.70 ± 0.02
D_0.__5_, µm	238 ± 2.38	11.9 ± 0.12
D_0.__9_, µm	392 ± 3.92	60.9 ± 0.61
D_32_, µm	202 ± 2.02	3.57 ± 0.036
D_43_, µm	253 ± 2.53	23.8 ± 0.24

D_0.1_—the diameter below which 10% of the particles lay; D_0.5_—the diameter below which 50% of the particles lay; D_0.9_—the diameter below which 90% of the particles lay; D_32_—the surface-based mean diameter; D_43_—the volume-based mean diameter.

**Table 2:** The composition of the initial and composite powders.

Composite	Nominal Powder Composition	Notes
nAl	nAl100 (Al, Al_2_O_3_)	Initial powder, air-passivated, 100 nm (nominal size)
nAl-H10	nAl100 (90 wt.%) + HTPB (10 wt.%)	Acetylacetone: 0.5 wt.% of nAl100 mass
nAl-H20	nAl100 (80 wt.%) + HTPB (20 wt.%)	Acetylacetone: 0.5 wt.% of nAl100 mass

**Table 3:** Base composition of the propellants tested.

Ingredients	Mass Fraction, wt.%
AP (coarse D_nominal_ = 200 µm)	65
AP (fine D_0.5_ = 18 µm)	10
HTPB	17
nAl	8

**Table 4:** Active aluminum content in the tested nAl powders.

Powder	C_Al_, wt.%	C_Al, Expected_^a^, wt.%
nAl100	85.9 ± 0.8	-
nAl100-H10	74.7 ± 0.5	77.3
nAl100-H20	63.4 ± 2.8	68.7

^a^ The expected aluminum content is estimated based on the nAl100.

**Table 5:** Burning rate of the formulations tested.

Formulations	a_r_, mm/(s bar ^nr^)	n_r_	R^2^	r_b_ (40 bar), mm/s
AP_nAl100	1.444 ± 0.045	0.531 ± 0.012	0.997	10.2 ± 0.2
AP_nAl100-H10	1.856 ± 0.027	0.483 ± 0.005	0.999	11.0 ± 0.1
AP_nAl100-H20	1.636 ± 0.080	0.527 ± 0.018	0.992	11.3 ± 0.5
